# Supplementary material for: Elastically induced phase-shift and birefringence in optical fibers
Source: Open Res Eur. 2025 Aug 13;5:99. Originally published 2025 Apr 7. [Version 2] doi: 10.12688/openreseurope.19414.2 (PMC12203310; doi:10.12688/openreseurope.19414.2)
Supplement: Peer review history

# Reviewer report: Reviewer response for version 2

**DOI:** 10.21956/openreseurope.22625.r58210

**Published:** 2025-09-10

**Authors:** Matthew Maitra

**Affiliations:** 1 ETH Zurich, Zürich, Zurich, Switzerland

I consider the revisions to be appropriate.

Is the study design appropriate and does the work have academic merit?

Yes

Is the work clearly and accurately presented and does it cite the current literature?

Partly

If applicable, is the statistical analysis and its interpretation appropriate?

Not applicable

Are all the source data underlying the results available to ensure full reproducibility?

No source data required

Are the conclusions drawn adequately supported by the results?

Yes

Are sufficient details of methods and analysis provided to allow replication by others?

Yes

Reviewer Expertise:

Continuum mechanics; numerical methods for PDEs; some expertise in general relativity

I confirm that I have read this submission and believe that I have an appropriate level of expertise to confirm that it is of an acceptable scientific standard.

# Reviewer report: Reviewer response for version 2

**DOI:** 10.21956/openreseurope.22625.r58209

**Published:** 2025-08-29

**Authors:** M. S. Mani Rajan

**Affiliations:** 1 Anna University, Ramanathapuram, India

1. Typo error in the Eqn (2.29)

 2. In birefringence optical fibers, there is always more than one mode propagation is possible. This means that theoretical representation of multi mode propagation is can be described by coupled equation. But why authors didn't use coupled systems.

 3. Importance of GRAVITES experiment should be discussed.

Is the study design appropriate and does the work have academic merit?

Yes

Is the work clearly and accurately presented and does it cite the current literature?

Partly

If applicable, is the statistical analysis and its interpretation appropriate?

Not applicable

Are all the source data underlying the results available to ensure full reproducibility?

No source data required

Are the conclusions drawn adequately supported by the results?

Partly

Are sufficient details of methods and analysis provided to allow replication by others?

Partly

Reviewer Expertise:

Nonlinear Fiber Optics and Fiber sensor

I confirm that I have read this submission and believe that I have an appropriate level of expertise to confirm that it is of an acceptable scientific standard.

# Reviewer report: Reviewer response for version 1

**DOI:** 10.21956/openreseurope.21009.r53942

**Published:** 2025-06-25

**Authors:** Matthew Maitra

**Affiliations:** 1 ETH Zurich, Zürich, Zurich, Switzerland

**SUMMARY**

 Steininger, Mieling and Chruściel derive the phase-shifts on photons passing through optical fibres subject to small elastic deformations. They do so via a multiple-scale perturbative scheme. They then use the results to discuss the associated systematic effects on the upcoming GRAVITES experiment.

**DISCUSSION**

 The manuscript is well-written, providing good explanation of the methods employed. I have not gone through every line of algebra (!) but it is clear from the text that the authors are in command of the theory (note also that they have already published multiple methodological ingredients of the present manuscript). Section 2 in particular is exceptionally clearly written -- not an easy feat given how fiddly multiple-scale analysis can get. Section 3 also gives a good discussion of the derivation of the terms needed within Section 2's final expressions (with a couple of caveats discussed below). Similarly, Tables 3.2 and 3.3 give a good overview of the implications for GRAVITES, subject to some caveats.

 1: Transformed interface terms (Section 3)

 Section 3's discussion of elasticity is conceptually sound. But in general, Section 3.1.1 needs to draw the same crystal-clear distinction between physical and referential quantities that is drawn in the preceding subsubsection on ``The equations''. See the following.

 1a

 I find eq.(3.27) to be misleading. It would be much clearer to me if \chi, A and G all had overbars and different brackets were used. This would help to emphasise that these are the conditions at the *physical* core-cladding interface, as distinct from the reference interface r = \rho that the physical conditions are transformed onto.

 For what it's worth, I would suggest that quantities associated with the zeroth-order reference have circle-diacritics while exact, physical quantities are simply unadorned. That would mesh well with Section 2.

 1b

 The wording around eq.(3.29) is a little awkward. \overline{A}_{\mu} is continuous across the *physical* interface, and since the cladding and core only differ in their optical properties (Table 3.2) there should be no discontinuity in the elastic deformation across the reference interface. From eq.(3.17) it then follows directly that A_{\mu} *is* continuous; this is not an assumption. (Unless the assumption is that the core and cladding have identical elastic properties; if so then please make explicit.)

 1c (tangential remark on layout)

 In section 3.1 we currently have ``The equations'' without a subsection heading, followed by ``Boundary conditions'' as Section 3.1.1. This seems excessively asymmetrical.

 2: Implications for GRAVITES

 2a

 From the last sentence of the first paragraph of Section 3.3, ``the response of the system is linear in the environmental parameters, [so] these numbers can directly be used to determine the constraints on the experimental setup so that environmental fluctuations do not overwhelm the signal of interest.'' Now, if I interpret Table 3.3 correctly, then the overall temperature-induced phase-shift is O(10^{8}) times the gravitational phase-shift of interest, while the overall pressure-induced phase shift is O(10^{6}) larger, with \delta T and \delta p as given in Table 3.2. For the systematic errors related to temperature and pressure each to be \approx 10% of the interesting signal, it would therefore seem that GRAVITES would need \delta T \approx 10^{-11}K and \delta P \approx 10^{-6}Pa. 

 Is this feasible? Not being an experimentalist I have no feel for this. If there is one message that this paper should communicate to any reader, it is surely the implication for GRAVITES of the paper's hard analysis. Therefore I would very much like to see a discussion along the lines of the previous paragraph. It is too important not to state explicitly.

 2b (tangential comment)

 When I reviewed the previous paper in this series (ref. [6]) I had some misgivings about approximating the fibre as straight, but now that I see how tiny a relative effect the ``Geometric deformation'' has on the phase shift, those misgivings are basically gone.

 CONCLUSION

 I would be glad to see this interesting work indexed subject to adequate treatment of the points mentioned above.

Is the study design appropriate and does the work have academic merit?

Yes

Is the work clearly and accurately presented and does it cite the current literature?

Partly

If applicable, is the statistical analysis and its interpretation appropriate?

Not applicable

Are all the source data underlying the results available to ensure full reproducibility?

No source data required

Are the conclusions drawn adequately supported by the results?

Yes

Are sufficient details of methods and analysis provided to allow replication by others?

Yes

Reviewer Expertise:

Continuum mechanics; numerical methods for PDEs; some expertise in general relativity

I confirm that I have read this submission and believe that I have an appropriate level of expertise to confirm that it is of an acceptable scientific standard, however I have significant reservations, as outlined above.

# Reviewer report: Reviewer response for version 1

**DOI:** 10.21956/openreseurope.21009.r53941

**Published:** 2025-06-02

**Authors:** Xiaohui Li

**Affiliations:** 1 Shaanxi Normal University, Xi'an, Shaanxi, China

This paper investigates the phase shifts and birefringence phenomena in optical fibers under elastic deformation (e.g., due to temperature, pressure, and gravity variations). Through theoretical analysis and numerical calculations, the authors derive the light propagation characteristics of fibers under elastic deformation and provide specific constraints for environmental control in fiber interferometers. The results demonstrate that temperature and pressure changes primarily affect the optical performance of fibers through elastic deformation and the photoelastic effect, while gravity gradients induce phase shifts and birefringence. These findings are significant for improving the precision and reliability of fiber interferometers, particularly in long-baseline fiber interferometry and gravitational experiments. Below are some suggestions for the paper:

1. The authors are advised to compare their work with recent related studies, such as *Adv. Opt. Mater.* **10**(24), 2201812 (2022); *Optics & Laser Technology*, **151**, 108016 (2022); *Optics & Laser Technology*, **158**, Part B, 108898 (2023); *Ultrafast Science*, **2022**, 9767251, 16 (2022); *Ultrafast Science*, **2022**, 9870325, 6 (2022); *Advanced Photonics*, **2**(2), 024001 (2020); *Advanced Photonics*, **2**(1), 015001 (2020). Such comparisons would help readers better understand the innovations and contributions of this work.
2. The paper assumes the fiber is an ideal linear isotropic medium, which simplifies the problem to some extent. However, real fibers may exhibit nonlinear effects and anisotropy. It is recommended to discuss the limitations of these assumptions and explore how nonlinearity and anisotropy might influence the results. Future work could incorporate these effects to provide a more comprehensive description of the fiber's physical properties.
3. The paper mentions that temperature-induced phase shifts in fibers are primarily manifested through elastic deformation but does not fully address the thermo-optic effect. It is suggested that the discussion be expanded to include the direct impact of temperature changes on the fiber's refractive index, thereby providing a more complete assessment of temperature effects on fiber interferometers. References [24] and [25] could be cited to further analyze the contribution of temperature variations to refractive index changes and compare them with elastic effects.
4. The paper presents abundant numerical results, but their physical significance could be further analyzed. For instance, the impact of phase shifts and birefringence under different environmental variations on the measurement accuracy of fiber interferometers could be discussed in greater detail, along with potential experimental methods to compensate for or correct these effects. Additional figures or graphs illustrating the trends of phase shifts and birefringence under varying environmental parameters could enhance readers' intuitive understanding of the results.
5. The introduction provides an overview of the research background and motivation, but this section could be strengthened. A more detailed explanation of why studying phase shifts and birefringence in fibers under elastic deformation is crucial for fiber interferometer applications—particularly in quantum optics and gravitational experiments—would be beneficial. Citing more experimental studies to demonstrate the practical implications of these elastic effects could further contextualize the research and reinforce its significance.

Is the study design appropriate and does the work have academic merit?

Yes

Is the work clearly and accurately presented and does it cite the current literature?

Yes

If applicable, is the statistical analysis and its interpretation appropriate?

Yes

Are all the source data underlying the results available to ensure full reproducibility?

Partly

Are the conclusions drawn adequately supported by the results?

Yes

Are sufficient details of methods and analysis provided to allow replication by others?

Yes

Reviewer Expertise:

This paper investigates the phase shifts and birefringence phenomena in optical fibers under elastic deformation (e.g., due to temperature, pressure, and gravity variations). Through theoretical analysis and numerical calculations, the authors derive the light propagation characteristics of fibers under elastic deformation and provide specific constraints for environmental control in fiber interferometers. The results demonstrate that temperature and pressure changes primarily affect the optical performance of fibers through elastic deformation and the photoelastic effect, while gravity gradients induce phase shifts and birefringence. These findings are significant for improving the precision and reliability of fiber interferometers, particularly in long-baseline fiber interferometry and gravitational experiments. Below are some suggestions for the paper:1.      The authors are advised to compare their work with recent related studies, such as Adv. Opt. Mater. 10(24), 2201812 (2022); Optics & Laser Technology, 151, 108016 (2022); Optics & Laser Technology, 158, Part B, 108898 (2023); Ultrafast Science, 2022, 9767251, 16 (2022); Ultrafast Science, 2022, 9870325, 6 (2022); Advanced Photonics, 2(2), 024001 (2020); Advanced Photonics, 2(1), 015001 (2020). Such comparisons would help readers better understand the innovations and contributions of this work.2.      The paper assumes the fiber is an ideal linear isotropic medium, which simplifies the problem to some extent. However, real fibers may exhibit nonlinear effects and anisotropy. It is recommended to discuss the limitations of these assumptions and explore how nonlinearity and anisotropy might influence the results. Future work could incorporate these effects to provide a more comprehensive description of the fiber's physical properties.3.      The paper mentions that temperature-induced phase shifts in fibers are primarily manifested through elastic deformation but does not fully address the thermo-optic effect. It is suggested that the discussion be expanded to include the direct impact of temperature changes on the fiber's refractive index, thereby providing a more complete assessment of temperature effects on fiber interferometers. References [24] and [25] could be cited to further analyze the contribution of temperature variations to refractive index changes and compare them with elastic effects.4.      The paper presents abundant numerical results, but their physical significance could be further analyzed. For instance, the impact of phase shifts and birefringence under different environmental variations on the measurement accuracy of fiber interferometers could be discussed in greater detail, along with potential experimental methods to compensate for or correct these effects. Additional figures or graphs illustrating the trends of phase shifts and birefringence under varying environmental parameters could enhance readers' intuitive understanding of the results.5.      The introduction provides an overview of the research background and motivation, but this section could be strengthened. A more detailed explanation of why studying phase shifts and birefringence in fibers under elastic deformation is crucial for fiber interferometer applications—particularly in quantum optics and gravitational experiments—would be beneficial. Citing more experimental studies to demonstrate the practical implications of these elastic effects could further contextualize the research and reinforce its significance.

I confirm that I have read this submission and believe that I have an appropriate level of expertise to confirm that it is of an acceptable scientific standard.

## References

[1] : Elastically induced phase-shift and birefringence in optical fibers. *Open Research Europe* .2025;5: 10.12688/openreseurope.19414.1 10.12688/openreseurope.19414.1 40584914 PMC12203310

# Reviewer report: Reviewer response for version 1

**DOI:** 10.21956/openreseurope.21009.r53003

**Published:** 2025-04-23

**Authors:** M. S. Mani Rajan

**Affiliations:** 1 Anna University, Ramanathapuram, India

Authors should reply to the following questions and improve the manuscript

 1. Why authors consider the ideal optical fibers?

 2. Typo error in many places. Example in Eqn. (2.29). Kindly check all equations. 

 3. Motivation of problem is not stated properly.

 4.  Why authors consider the linear media?. Optical fiber is a nonlinear medium. Justify. 

 5. Why authors describe dispersion less medium?.

Is the study design appropriate and does the work have academic merit?

Yes

Is the work clearly and accurately presented and does it cite the current literature?

Partly

If applicable, is the statistical analysis and its interpretation appropriate?

Not applicable

Are all the source data underlying the results available to ensure full reproducibility?

No source data required

Are the conclusions drawn adequately supported by the results?

Partly

Are sufficient details of methods and analysis provided to allow replication by others?

Partly

Reviewer Expertise:

Nonlinear Fiber Optics and Fiber sensor

I confirm that I have read this submission and believe that I have an appropriate level of expertise to confirm that it is of an acceptable scientific standard, however I have significant reservations, as outlined above.
